# Westminster Hospital

**Published:** 1830-07

**Authors:** 


### HOSPITAL REPORTS.

### WESTMINSTER HOSPITAL.

### *Cases of Popliteal Aneurism.*

Case I. William Cooper, æt. twenty-six, admitted March 24th, under the care of Mr. Guthrie. Is a farmer's labourer, residing at Leatherhead, of middling stature, moderately muscular. He states that he has enjoyed good health until four months since, when, whilst walking, he experienced suddenly a sensation like that of cramp in the left ham. This feeling occasionally recurred, but was not sufficient to prevent him from following his usual avocations. About a month since he became conscious of pulsation in the part, but still he did not desist from his labours. This symptom became aggravated, and he consulted a professional man, who, considering it a case of popliteal aneurism, advised him to enter an hospital. Of his own accord he applied a bandage to the part, which was rather hurtful than otherwise.

Present state: The thorax apparently well formed; percussion exhibits a laudable sound through all parts of the chest, which expands fully on inspiration; respiratory murmur natural; action of the heart regular; systole of the auricles and ventricles perfectly distinct, both in the left intercostal space and at the base of the sternum; the force of the heart is much increased, and its sound is perceptible all over the anterior part of the chest and in the axillary regions. The patient is in other respects perfectly healthy.

A tumor exists in the left popliteal space, making a considerable prominence at the seat of flexion: this may be made to disappear by steady pressure, and it has a pulsation synchronous with that of the artery, and equally distinct on all parts of the tumor; pressure on the femoral artery in the groin removes the pulsation entirely. The patient complains of a dull pain in the part, extending to the calf of the leg; and the affected leg is considerably larger than the other, and slightly œdematous.—Ordered low diet, beef-tea and one egg daily. To be kept quiet in bed.

March 28th.—The bowels have been kept open by house medicine. Was bled yesterday to eight ounces; the blood abstracted was slightly buffed and cupped; on which account, Mr. Guthrie postponed the operation till April 10th.

30th.—V.S. ad ℥xiv. The appearance of the blood is nearly natural.

April 4th.—Much the same; pulse seventy-two, soft, and tolerably full.—Ordered to be bled to ℥xij.

5th.—The blood drawn yesterday is slightly buffed, but not so much as before. The tumor is gradually increasing in size, and a dull pain still continues in his leg, which is rather worse at night. General health good; bowels open, tongue whitish. The patient is very anxious to have the operation performed.

9th.—Mr. Guthrie now directed that the temperature of both legs should be ascertained, which was done by the aid of a small thermometer.

Ten A.M., temperature of the right leg 92°, of the left leg 92°.
Ten P.M. 96°, 96°.

10th.—At one o'clock A.M., the operation of tying the femoral artery in the upper third of the thigh was performed. In the evening, after the operation, he complained of shooting pains in the knee and calf of the leg; pulse full. New flannel was now applied to the whole limb, with bottles of hot water to the foot.

Four P.M., temperature of the right leg 86°, left leg 82°.

Ten P.M.—The patient has dozed a little; pain not so severe; leg feels of a natural heat; pulse eighty, rather full. Temperature of the right leg 93°, left leg 91°.

11th, eight A.M.—Was very comfortable during the night. This morning he complains to Mr. Guthrie of pain in the seat of the incision. The leg is apparently more swollen; veins slightly distended: no pulsation in the tumor; temperature of both legs 92°; pulse 100; tongue furred and whitish; skin hot; pain in the head. Bowels have not been relieved since the operation. Mr. Guthrie ordered the following mixture: ℞. Potass. Carb. ℈i.; Suc. Limon, ʒiij.; Aquæ ʒx.; Sodæ Sulph. ʒi. M. fiat haust. quartis horis sumendus.

Six P.M.—Bowels have not acted. Pain in the head better; pulse 100, offering some slight resistance to the finger; temperature of the right leg 98°, left leg 96°. Ordered by Mr. Guthrie to take Hydrarg. Submur. gr. iv.; Extract. Col. comp. gr. vi. in pil. Mist. Salin. contin. secundis horis donec alvus soluta fuerit.

Eleven P.M.—Bowels not yet moved. Capiat Ol. Ricini ℥i. statim, et repetatur secundâ quâque horâ si opus sit.

12th, eight A.M.—Was seen by Mr. Guthrie. The patient had two scanty evacuations in the night, and he slept tolerably well. His skin is not so hot; pulse as frequent; has slight shooting pains down the inner part of the thigh. Temperature of both legs 94°.—V.S. ad ℥vi. Haustus aperiens statim.

Ten A.M.—Has passed three evacuations. Pain in the head relieved: he has slight pain in the course of the femoral vein; countenance less flushed; pulse 110, irritable. Blood abstracted this morning is slightly buffed, but not cupped. Says that he feels low and weak. Mr. Guthrie ordered the patient to take Mistura Salin. cum Liq. Opii Sedat. ♏iij. quartis horis.

Half-past six P.M.—Much better. Pains in the thigh not so frequent; countenance more cheerful; skin cool; pulse 110; tongue moist; bowels freely open.—Cont. med.

Ten P.M.—Has suffered from slight pains in the abdomen; limb quite easy. To take Liq. Opii Sedat. ♏iv. directly, and to repeat the dose at twelve o'clock, if it be necessary. The night nurse ordered to report the state of the patient every four hours.

13th, eight A.M.—The draught was repeated at twelve o'clock, and the patient slept the remaining part of the night. He is in no pain at present, but occasionally experiences shooting pains in the course of the femoral vein. Just below the incision there appears a slight blush of redness, with fulness and tenderness on pressure. Temperature of both legs 93°; pulse ninety-eight, and soft; tongue not so much furred, and moist. The diseased leg is not so much swollen; and Mr. Guthrie thought the tumor not so large as it was previous to the operation. The strips of adhesive plaster were loosened, and the patient ordered to take some opening medicine. Twelve leeches to be applied in the course of the femoral vein, and a bread and water poultice afterwards.

Four P.M.—The leeches have bled freely, and he feels some slight relief. In other respects much the same; pulse 110.—To be bled at nine o'clock.

Ten P.M.—Pulse 120, full and hard. The patient was now bled to ℥xij., and he expressed himself much relieved.—Cont. Liq. Opii Sedativus.

14th, eight A.M.—Has passed a good night, and this morning he has but very little pain in the thigh. The blood is buffed and cupped. Pulse 810, soft; skin cool; tongue white; bowels freely open; temperature of both legs 92°.—Rep. med.

Ten A.M.—The strips of adhesive plaster were removed: the wound appeared healing; a good deal of healthy matter discharged, which was pressed out, and the wound dressed. There is slight tenderness of the thigh, but the patient suffers very little pain; no pulsation of the tumor; countenance slightly blanched, but cheerful.—Rep. med.

Ten P.M.—The thigh much easier since the morning.—Capiat Liq. Opii Sedativ. ♏iv.

15th.—The patient slept well last night. There is slight tenderness on pressure at the lower part of the abdomen, and he complains of an inability to pass his urine. No pain in the thigh; great discharge from the wound; skin cool and moist; pulse eighty-two, soft; tongue clean, bowels open.

Ten A.M.—Has voided his urine, and he now feels much relieved. A roller with padding was applied in the course of the femoral vessels, to prevent the lodgment of matter.

16th.—The wound was dressed this morning. The patient is going on well; pulse soft; has no pain in any part.

17th.—Dressed by Mr. Guthrie, who observed that the tumor is much smaller. The veins of the leg are less distended.

27th.—The patient has continued gradually improving, the wound being dressed every morning, and the bowels regulated with small doses of Epsom salts. This morning both ligatures came away. Some luxuriant granulations were checked by an application of the lunar caustic, which produced some swelling and tenderness: these were speedily relieved by the aid of a poultice.

May 16th.—Since the last report, the patient's general health has progressively improved. A slight purulent discharge existed for several days, but it has now entirely ceased, and the cure may be considered complete.

Case II. Sunday, May 16th, 1830.—Thomas M'Caughin, æt. thirty-three, admitted May 14th, with popliteal aneurism in the left leg. He was brought up as a gardener, and entered the army at the age of seventeen. States that his health has never been affected. He is about five feet ten inches high. On the 2d of April, whilst making a charge on a field day, his leg was crushed; the holster of his left-hand comrade struck him in the ham; the whole limb, from the knee downwards, swelled, and became very painful; there was no discoloration of integuments, and he continued his duty for about four days after the accident. The day after the receipt of the injury, whilst marching, he first perceived a pulsation in the left ham, which continued to increase until, on passing through London, he was received into hospital.

The patient appears to be in sound health; his chest is well formed, broad, and capacious; respiratory murmur natural, percussion elicits no peculiar sound; action of the heart regular; pulse sixty-four; bowels open; tongue clean; appetite unimpaired. Temperature of the limb 88°.—Ordered to bed, and to have low diet.

On Saturday, May 15th, Mr. Guthrie, in the presence of the other medical officers and a numerous class of pupils, secured the femoral artery at the upper third of the thigh. After the patient had been removed to his ward, Mr. Guthrie directed that the limb should be placed on pillows in the half-flexed position, and flannel to be wrapped around it. If the temperature should get lower than natural, bottles of hot water are to be applied to the soles of the feet.

Three P.M.—The temperature of the limb, about an hour and a half after the operation, was 88°. He is very comfortable, and at present free from pain.

Five P.M.—Was seen by Mr. Guthrie, who remarked that the tumor was somewhat diminished in size already. The veins of the leg were fuller than they were previous to the operation; temperature of the limb 88°. Towards the evening complained of sickness from the constant pain around the thigh and ankle-joint. Does not experience the sensation of numbness of the leg.—Capiat Liq. Opii Sedativ. ♏xv.

May 16th, eight A.M.—Was visited by Mr. Guthrie. Has passed a comfortable night; no febrile excitement; thigh and leg easy; pulse sixty-eight; bowels not yet opened; tongue moist; skin natural; countenance cheerful.

Ten P.M.—Feels rather sick; bowels have not been relieved since the operation; complains likewise that he is unable to void his urine; no pain, but a sensation of fulness in the pudic region; tongue moist, and covered with a whitish fur; pulse seventy-four, slightly accelerated. No pain in the thigh or leg; temperature of the limb 92°.

17th, eight A.M.—Has passed a comfortable night: sickness has subsided; bowels have been freely acted upon; tongue moist and whitish; no thirst; skin moist; pulse seventy. Complains of a sensation of numbness in the foot. Temperature of the left leg 93°, right 92°.

Nine P.M.—Feels comfortable: no pain in the leg or thigh. Bowels open seven or eight times, in consequence of the night nurse administering the medicine rather too freely. Sensation of numbness continues. Temperature of both limbs 93°.

18th.—Visited by Mr. Guthrie. Has passed a good night: no pain in the leg or thigh; bowels open; tongue moist, and covered with a whitish fur; skin cool; pulse seventy-four. The temperature of the limb operated upon is 92°. Does not take any medicine. Wound has not been yet dressed.

19th.—Wound dressed, having nearly healed by the first intention.

The ligature remained until the 31st day, when it came away spontaneously. This patient never had a bad symptom.

Mr. Guthrie performed these operations with that coolness for which he is remarkable, demonstrating the parts as they were divided, as if it had been a dissection on the dead body, in a manner most advantageous for the students. The patients suffered but little pain.

